# Up-regulation of IGF2BP2 by multiple mechanisms in pancreatic cancer promotes cancer proliferation by activating the PI3K/Akt signaling pathway

**DOI:** 10.1186/s13046-019-1470-y

**Published:** 2019-12-18

**Authors:** Xiaodong Xu, Yan Yu, Ke Zong, Pengwei Lv, Yuantin Gu

**Affiliations:** 1 Department of Breast Surgery, The First Affiliated Hospital of Zhengzhou University, No.1 Jianshe east Road, Zhengzhou, 450000 China; 2 Department of Infection Disease, The First Affiliated Hospital of Zhengzhou University, Zhengzhou, 450000 China; 3 Department of Hepatobiliary and Pancreatic Surgery, The First Affiliated Hospital of Zhengzhou University, Zhengzhou, 450000 China

**Keywords:** Pancreatic cancer, IGF2BP2, Genomic amplification, miR-141, PI3K/Akt pathway, Proliferation

## Abstract

**Background:**

The survival of pancreatic cancer patients remains poor. However, the underlying molecular mechanism and new therapeutic target of pancreatic cancer are still needed to be found. Many studies have shown that the IGF2 mRNA-binding protein 2 (IGF2BP2) plays oncogenic roles in cancers. However, the clinical significance, role and molecular mechanisms of IGF2BP2 in pancreatic cancer remain unclear.

**Methods:**

The expression of IGF2BP2 and miR-141 was detected in pancreatic cancer, and clinical significances were analyzed by statistical analysis. The function of IGF2BP2 and miR-141 was determined in vitro and in vivo, and the underlying mechanism was investigated. The gene copy number variation (CNV) of IGF2BP2 was analyzed based on The Cancer Genome Atlas (TCGA) dataset. microRNAs (miRNAs) regulating IGF2BP2 were predicted by online tools and confirmed by experiments.

**Results:**

IGF2BP2 is overexpressed in pancreatic cancer tissues compared with control tissues. Upregulation of IGF2BP2 predicts shorter overall survival (OS) in pancreatic cancer patients by statistical analysis. IGF2BP2 overexpression is partially due to genomic amplification. Bioinformatics analyses and validation experiments showed that IGF2BP2 is a direct target of miR-141. A negative correlation between IGF2BP2 mRNA expression and the expression of miR-141 was observed in pancreatic cancer tissues and more importantly, reexpression of miR-141 rescued the oncogenic role of IGF2BP2. Moreover, upregulating IGF2BP2 expression promotes pancreatic cancer cell growth by activating the PI3K/Akt signaling pathway in vitro and in vivo.

**Conclusions:**

We comprehensively reveal the oncogenic role of IGF2BP2 in pancreatic cancer carcinogenesis and confirm that genomic amplification and the silencing of miR-141 contribute to its activation. Our findings highlight that IGF2BP2 may be a promising molecular target for the treatment of pancreatic cancer.

## Background

Pancreatic ductal adenocarcinoma (PDAC) is one of the most prevalent malignancies and remains a major public health problem worldwide [1]. The poor clinical outcomes of pancreatic cancer are due to lack of effective treatments, tumor metastasis and recurrence, as well as chemoresistance [2–5]. Genetic and epigenetic aberrations are frequently found in pancreatic cancer and associated with the aberrant activation of tumor driver genes [6, 7]. Therefore, it is of great importance to identify novel oncogenes involved in the tumorigenesis of pancreatic cancer and improve the overall survival of pancreatic cancer patients.

IGF2BP2, a member of the conserved IGF2 mRNA-binding protein family, regulates subcellular mRNA localization, stability and translation [8, 9]. Studies have shown that IGF2BP2 is overexpressed and promotes tumor progression in a variety of cancers, such as glioblastomas multiforme and gallbladder cancer [10, 11]. Further studies have showed that IGF2BP2 regulates the translation of IGF2 and increases the PI3K/Akt signaling pathway activation [12]. However, the role of IGF2BP2 in pancreatic cancer has not been established.

microRNAs (miRNAs) downregulate the expression of target genes by binding to the 3′-untranslated regions (UTRs) of target mRNAs [13]. miRNAs are involved in a wide range of cellular processes [14, 15]. Abnormal expression of miRNAs have been observed to be correlated with oncogenesis in human cancers. Moreover, as a member of the miR-200 family, miR-141 has been reported to be downregulated and correlated with malignancy initiation, progression and metastasis in certain cancers [16–18]. However, the role of miR-141 in pancreatic cancer remains to be elucidated.

In this study, we found that IGF2BP2 was upregulated in pancreatic cancer and promoted tumor cell proliferation through the PI3K-Akt pathway. The abnormal overexpression of IGF2BP2 is proposed to be partly due to genomic amplification and posttranscriptional regulation by tumor suppressor miRNA-141. The clinical correlation and survival prediction analysis revealed that IGF2BP2 is a promising and reliable prognostic marker for pancreatic cancer patients. Collectively, our findings suggest that IGF2BP2 upregulation is vital for the carcinogenesis of PDAC and might be a potential therapeutic target.

## Materials and methods

### PDAC cell lines and tissues

Six pancreatic cancer cell lines (AsPC-1, BxPC-3, Canpan-2, Mia PaCa-2, Panc-1 and Pan 03.27) and human pancreatic ductal epithelial (HPDE) cells were purchased from the Cell Repository, Chinese Academy of Sciences (Shanghai, China). All cells were cultured as described in a previous study [5]. All cell lines in our study were authenticated by short tandem repeat DNA profiling within 3 months and eliminated the mycoplasma contamination. 60 pairs of Pancreatic cancer tissues and adjacent normal pancreas tissues were collected between February 2012 and February 2014 at the First Affiliated Hospital of Zhengzhou University. All 60 cancers were identified as adenocarcinomas histologically.

### RT-qPCR

TRIzol reagent (Invitrogen, Carlsbad, CA) was used to extract total RNA according to the manufacturer’s instructions. A High-Capacity cDNA Reverse Transcription Kits (Applied Biosystems, Carlsbad, CA) was used for reverse transcription according to the manufacturer’s instructions. The RT-qPCR assays of mRNA expression levels were performed using a SYBR Green PCR Kit according to the manufacturer’s instructions. Reaction conditions were as previously described [5]. The expression levels of miR-141 and IGF2BP2 were normalized to U6 and GAPDH expression, respectively. Relative expression was analyzed using the 2^−ΔΔct^ method. Relative sequences are listed in Additional file 1: Table S1.

### Western blot analysis

The primary antibodies used in this study were IG2BP2 (11601–1-AP; ProteinTech Group, Inc), Phospho-Akt (Ser473) (#4060, CST), GAPDH (#5174, CST), Akt (#4691, CST), cleaved PARP (#5625, CST), cleaved caspase-3 (#9664, CST), CDK2 (#2546, CST), p21 Waf1/Cip1 (#2947, CST) and Cyclin D1 (#2978, CST). The secondary antibodies and enhanced chemiluminescence reagent were purchased from Boster (Wuhan, China). The protein extraction and western blot analysis were performed as described in our previous study [5].

### Immunohistochemistry

Immunohistochemistry was performed as described in a previous study [15]. All staining was scored using a semiquantitative method according to the staining intensity and the positive rate. The staining intensity was determined as 0 = negative, 1 = weak, 2 = moderate, and 3 = strong. The positive rate was defined as 0, < 1%; 1, 1–25%; 2, 26–50%; 3, 51–75%; and 4, 75–100%. IHC scoring more than 6 was defined as high expression.

### Cell transfection

The siRNAs of IGF2BP2, miRNA precursor of miR-141 and control were purchased from RiboBio company (Guangzhou, China). Cells grown in 6-well plates were transfected with a final concentration of 50 nM. Lipofectamine 2000 (Invitrogen) was used for all transfection assays according to the manufacturer’s protocol. IGF2BP2 and miR-141 overexpression lentivirus were constructed and synthesized by GenePharm (Shanghai, China). Stable transfection cells were selected with puromycin for 2 weeks. The efficiency of all transfections was evaluated by RT-qPCR and western blot. Relative sequences are listed in Additional file 1: Table S1.

### Cell proliferation assay

Cell Counting Kit-8 (Dojindo Laboratories, Kumamoto, Japan) was used to measured cell viability according to the manufacturer’s instructions. Different groups of cells (2000 per well) were plated in 96-well plates. After culturing for 24 h, CCK-8 reagent was added and the cells were incubated at 37 °C for 2 h and the absorbance was measured at 450 nm.

### Cell apoptosis and cell cycle assays

After treatment, cells were stained using the annexin V/PI apoptosis kit (MultiSciences, Hangzhou, China) according to the manufacturer’s instructions. Annexin V+ cells were examined by flow cytometry (BD Biosciences, San Jose, CA, USA). For cell cycle analysis, cells were harvested and fixed with 70% cold ethanol at − 20 °C. Then, cells were stained using the Cell Cycle Kit (MultiSciences, Hangzhou, China) according to the manufacturer’s instructions. The cell cycle distribution was examined by flow cytometry (BD Biosciences, San Jose, CA, USA).

### Luciferase reporter assays

Wild-type (wt) and mutant (mut) 3′-UTR of IGF2BP2 were constructed by RiboBio company (Guangzhou, China). Pancreatic cancer cells were cotransfected with the luciferase constructs and miR-141 mimics according to the manufacturer’s protocol. After transfection for 48 h, luciferase activities were measured using a Dual-Luciferase Reporter Assay Kit (Promega). Each experiment was conducted in triplicate.

### In vivo tumorigenicity model

Female 6-week-old Balb/c nude mice were purchased from HFK Bioscience (Beijing, China) and bred in the Central Animal Laboratory of Zhengzhou University under pathogen-free conditions. 2 × 10^6^ treated Panc-1 cells (suspended in 0.1 ml of PBS) were injected subcutaneously into the right armpit of the nude mice. The weight and the tumor diameter of each mouse were measured every week. Tumor volume (mm^3^) was calculated as follows: (shortest diameter)^2^ × (longest diameter) × 0.5. 7 weeks later all mice were killed.

### Statistical analysis

All statistical analyses were performed by SPSS 17.0 software (IBM Corp., Armonk, NY). All graphs were generated using GraphPad Prism 6.0 software (GraphPad Software Inc., La Jolla, CA, USA). *P* <  0.05 was considered statistically significant. Expression correlation analysis was evaluated using the Pearson correlation coefficient. Survival analysis was evaluated by the Kaplan-Meier method and the log-rank test. Two-tailed student’s t test was used to compare the differences between two paired or unpaired groups. The Cox proportional hazards model was employed for identifying independent prognostic factors. Gene set enrichment analysis (GSEA) was conducted by the GSEA 3.0 software (Broad Institute, Cambridge, MA, USA).

## Results

### IGF2BP2 is highly expressed in pancreatic cancer

**Fig. 1:**
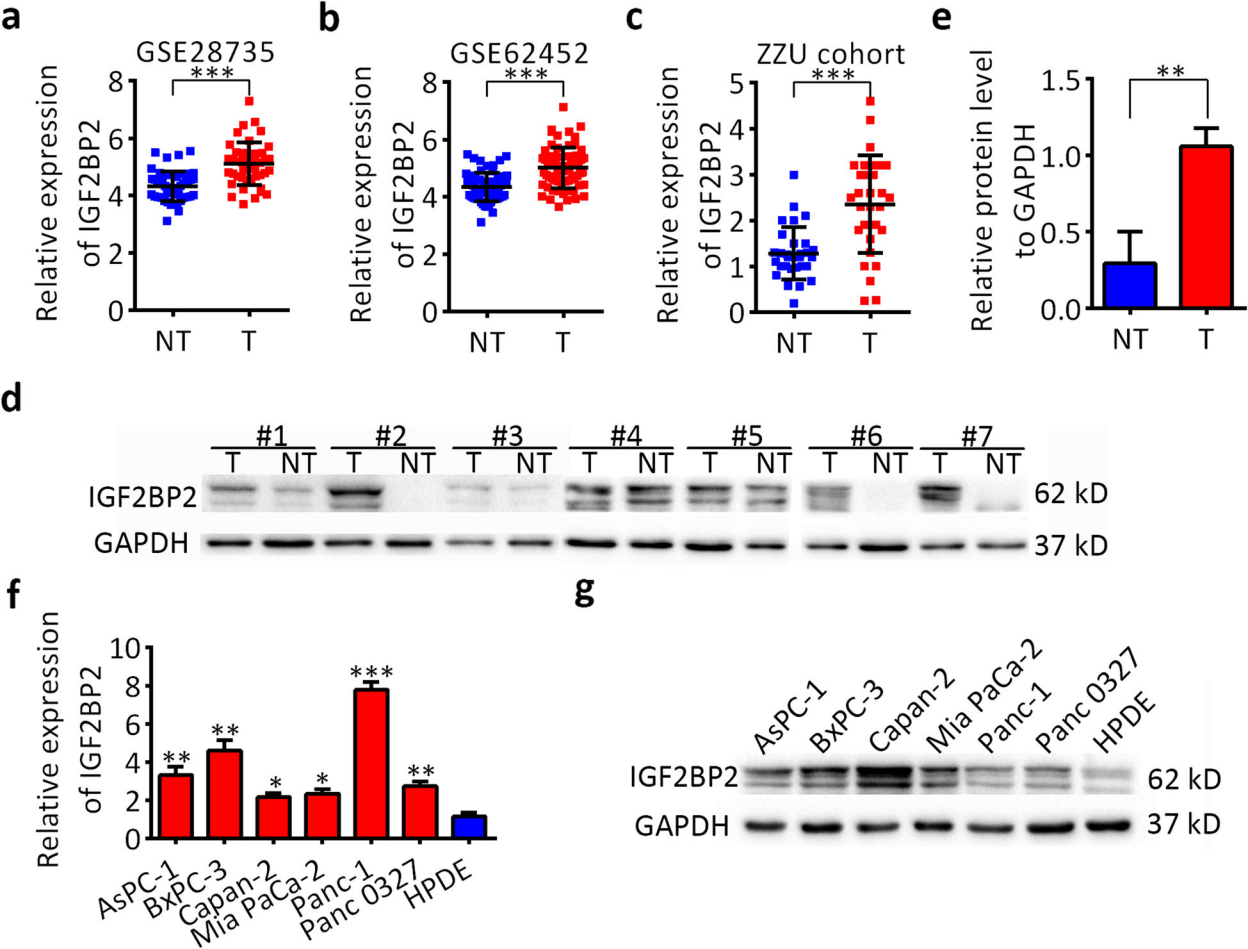
IGF2BP2 is upregulated in pancreatic cancer tissues and cell lines. **a** and **b** IGF2BP2 expression was analyzed in the PDAC GEO datasets GSE62452 and GSE62165. **c** RT-qPCR analysis of IGF2BP2 expression in pancreatic cancer tissues and adjacent normal tissues. **d** and **e** Western blot analysis of IGF2BP2 expression in pancreatic cancer tissues and adjacent normal tissues. **f** RT-qPCR analysis of IGF2BP2 expression in 6 PDAC cell lines and HPDE cells. **g** Western blot analyses of IGF2BP2 expression in 6 PDAC cell lines and HPDE cells. GAPDH served as an internal reference. Data are presented as the mean ± SD of at least three independent measurements. *P < 0.05, **P < 0.01 and ***P < 0.001. *P* < 0.05 was considered statistically significant

Data mining from Gene Expression Omnibus (GEO) datasets showed that IGF2BP2 was upregulated in pancreatic cancer tissues (T) compared with nontumor tissues (NT) (Fig. 1a,b). RT-qPCR results showed that IGF2BP2 mRNA was increased in human PDAC tissues compared with matched adjacent normal tissues, which were collected in the First Affiliated Hospital of Zhengzhou University (ZZU cohort; *n* = 30, Fig. 1c). In addition, western blot analysis showed that IGF2BP2 protein levels were also upregulated significantly in PDAC samples compared with those in matched adjacent normal tissues (*n* = 7, Fig. 1d,e). To verify this, we detected the expression levels of IGF2BP2 in pancreatic cancer cell lines by RT-qPCR and western blot. Compared with the normal pancreatic cell line HPDE, the mRNA and protein levels of IGF2BP2 were upregulated in all 6 tested pancreatic cancer lines (Fig. 1f,g). Taken together, these results indicate that IGF2BP2 is frequently upregulated in pancreatic cancer.

### Overexpression of IGF2BP2 predicts poor survival of pancreatic cancer patients

**Fig. 2:**
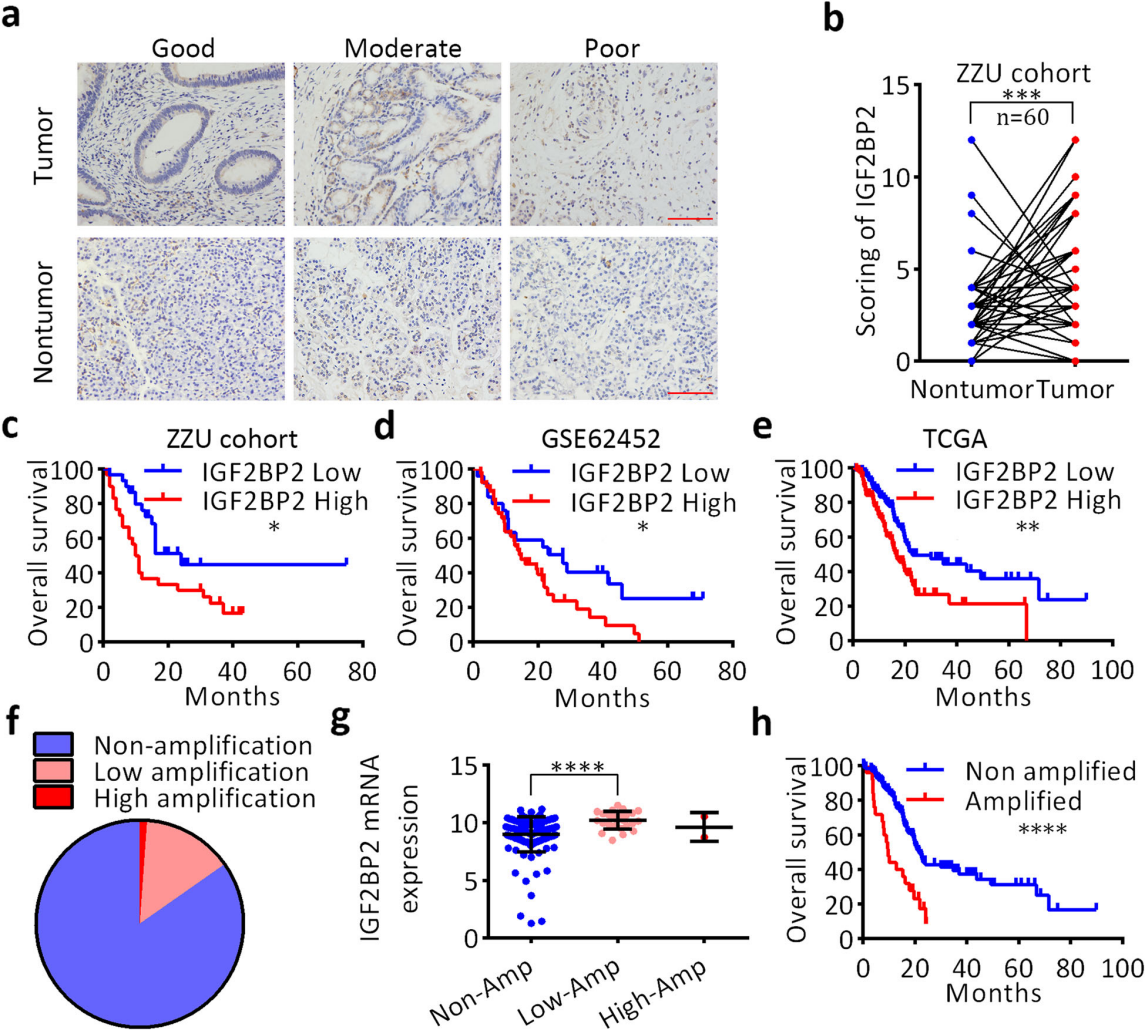
Overexpression of IGF2BP2 predicts poor survival of pancreatic cancer patients. **a** Representative images of IGF2BP2 staining in pancreatic cancer tissues of various differentiation statuses. Scale bar, 50 μm (red line). **b** Relative expression score of IGF2BP2 in pancreatic cancer by IHC. **c** Overexpression of IGF2BP2 was associated with poorer prognosis of PDAC patients of ZZU cohort. **d** Overexpression of IGF2BP2 was associated with poorer prognosis of PDAC patients in the GSE62452 dataset. **e** Overexpression of IGF2BP2 was associated with poorer prognosis of PDAC patients in the TCGA dataset. **f** Frequency (%) of IGF2BP2 genomic alterations in the TCGA cohort. **g** mRNA expression of IGF2BP2 (RNA Seq V2 RSEM)(log2) plotted against the putative copy-number alterations from GISTIC. **h** Patients with genomic alterations had poorer survival status. *P < 0.05, **P < 0.01, and ***P < 0.001. *P* < 0.05 was considered statistically significant

To investigate the clinical significance of IGF2BP2 in pancreatic cancer, IHC staining was performed on 60 pairs of pancreatic cancer tissues and matched nontumor tissues with complete clinicopathological and follow-up data available. Pancreatic cancer specimens with good, moderate and poor differentiation grades were stained for IGF2BP2, as shown in Fig. 2a. Quantitative analysis showed that IGF2BP2 was upregulated in pancreatic cancer tissues compared with paired nontumor tissues (Fig. 2b). Clinicopathological correlation analysis showed that upregulation of IGF2BP2 was correlated with tumor grade, TNM stage, and tumor size (Table 1). Kaplan–Meier analysis showed that increased IGF2BP2 levels predicted shorter overall survival (OS) time (median: 10.5 months vs. 24 months, respectively; log-rank test, *P* <  0.05; Fig. 2c). Importantly, Cox multivariate regression analysis showed that increased expression of IGF2BP2 could be an independent factor for prognosis (hazard ratio [HR] = 2.395, 95% confidence interval [CI] = 1.655–4.134; *P* < 0.05; Additional file 2: Table S2). In addition, Kaplan–Meier survival analysis of GSE62452 data also revealed that patients with high IGF2BP2 expression levels had shorter overall survival (median: 14.9 months vs. 27.7 months, respectively; log-rank test, *P* < 0.05; Fig. 2d). To further investigate the clinical implications of IGF2BP2 upregulation in pancreatic cancer, the correlation of the mRNA expression level of IGF2BP2 with clinicopathological features and survival status of patients in the TCGA dataset was analyzed (*n* = 171). Expression analysis showed that IGF2BP2 was generally expressed in PDAC tissues in the TCGA dataset (Additional file 5: Figure S1). IGF2BP2 was significantly overexpressed in cancerous tissues compared with the normal controls (*P* < 0.05, Additional file 6: Figure S2). Kaplan–Meier survival analysis showed that overexpression of IGF2BP2 predicted a poor survival status (median: 16.79 months vs. 22.83 months, respectively; log-rank test, *P* < 0.01; Fig. 2e). To further determine whether IGF2BP2 is an independent prognostic factor within the context of other clinical features, such as age, sex, neoplasm histological grade or TNM stage, a Cox multivariate regression analysis was performed. The results showed that IGF2BP2 maintained independence when predicting the OS of patients with pancreatic cancer (HR = 3.895, 95% CI = 1.895–4.634, *P* < 0.001; Additional file 3: Table S3). Then, we evaluated the predictive performance of IGF2BP2 by receiver operating characteristic (ROC) analysis, AUC values of 0.7540, 0.7864, and 0.7513 were found for predicting OS at 1, 2 and 3 years, respectively (Additional file 7: Figure S3). To investigate the mechanism contributing to the upregulation of IGF2BP2 in pancreatic cancer, genomic alterations analysis was performed using the cBio-Portal database. As demonstrated in Fig. 2f, IGF2BP2 locus was amplified in 15.25% of pancreatic cancer samples. Notably, IGF2BP2 mRNA expression level was significantly associated with IGF2BP2 CNV, indicating that genomic amplification may be one of the underlying mechanisms for the abnormal expression of IGF2BP2 in human pancreatic cancer (Fig. 2g). Kaplan–Meier survival analysis revealed that patients with IGF2BP2 genomic amplification showed poorer outcomes compared with patients without IGF2BP2 genomic amplification (median: 9.63 months vs. 21.71 months, respectively; log-rank test, *P* < 0.0001; Fig. 2h). Collectively, these findings demonstrat that IGF2BP2 is highly expressed in pancreatic cancer, is an independent predictor of prognosis and performs well in predicting the outcomes of patients with pancreatic cancer. Gene amplification is common in human pancreatic cancer and significantly contributes to the overexpression of IGF2BP2, which suggests that upregulation of IGF2BP2 might induce oncogenic effects in pancreatic cancer.

**Table 1:** Expression of IGF2BP2 according to clinicopathological features of 60 pancreatic cancer patients

Characteristic	High	Low	*P*-value
Age	31	29	0.586
≥ 65 years	16	17	
< 65 years	15	12	
Sex	32	28	0.63
Male	21	20	
Female	11	8	
Grade			0.034*
Good	2	3	
Moderate	19	10	
Poor	23	3	
TNM			0.02*
I&II	22	12	
III&IV	9	17	
Size			< 0.001*
≥ 2.5 cm	20	8	
< 2.5 cm	9	23	
CA199			0.342
≥ 100	33	10	
< 100	11	6	

*Statistical significant difference

### IGF2BP2 accelerates pancreatic cancer cell proliferation in vitro

**Fig. 3:**
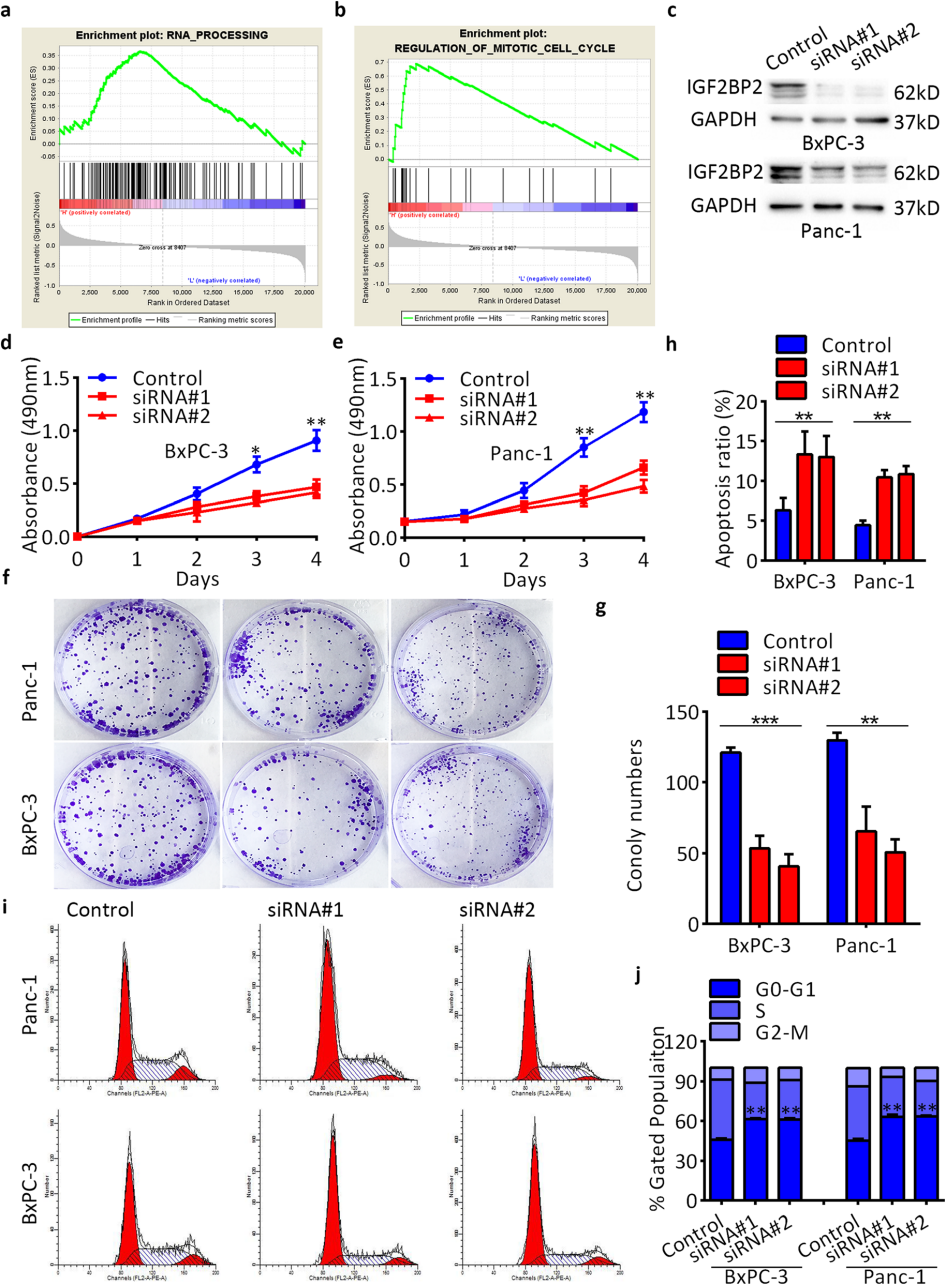
IGF2BP2 promotes pancreatic cancer proliferation of pancreatic cancer in vitro. **a-b** GSEA analysis comparing IGF2BP2 high-expression group (red) to the IGF2BP2 low-expression group (blue) of pancreatic cancer tissues. **c** IGF2BP2 protein levels after transfection analyzed by western blot assay. **d** and **e** CCK-8 assay of cell viability in BxPC-3 and Panc-1 cell lines after transfection. Cell viability was determined at 24, 48, 72 and 96 h. **f** and **g** Colony formation assay of cell viability in BxPC-3 and Panc-1 cell lines after transfection. **h** Flow cytometry assay of apoptosis of BxPC-3 and Panc-1 cells after transfection. **i** and **j** Flow cytometry assays of cell cycle distribution of BxPC-3 and Panc-1 cells after transfection. The percentage of cells in each cell cycle phase is shown in the inset of each panel. GAPDH served as a loading control. Data are represented as the mean ± SD of at least three independent measurements. *P < 0.05, ** *P* < 0.01, and ***P < 0.001. *P* < 0.05 was considered statistically significant

To understand the role of elevated IGF2BP2 in the carcinogenesis of pancreatic cancer, GSEA was performed in the published dataset NCBI/GEO/GSE62165 (*n* = 118). The results revealed that genes associated with the cell cycle and RNA processing were significantly enriched in IGF2BP2-overexpressing cases (Fig. 3a,b). To validate the biological effects of IGF2BP2 in pancreatic cancer, two specific siRNAs targeting IGF2BP2 were transfected into two IGF2BP2-overexpressing cells (BxPC-3 and Panc-1), and an unspecific siRNA was transfected as a negative control. The transfection efficency was confirmed by RT-qPCR and western blot assays (Fig. 3c and Additional file 8: Figure S4). Silencing of IGF2BP2 markedly inhibited the growth of both cells, as indicated by the CCK-8 assays (Fig. 3d,e) and colony formation assays (Fig. 3f,g). Then, flow cytometry assays were conducted to investigate the potential mechanism by which IGF2BP2 promotes pancreatic cancer cell proliferation. The results showed that depletion of IGF2BP2 resulted in an increase in the apoptosis ratio (Fig. 3h; Additional file 9: Figure S5) and cell cycle arrest in the G0/G1 phase (Fig. 3i,j). These results indicate that IGF2BP2 can induce the proliferation of pancreatic cancer cells by inducing cell cycle progression and inhibiting of cell apoptosis.

### IGF2BP2 is a direct target of miR-141

**Fig. 4:**
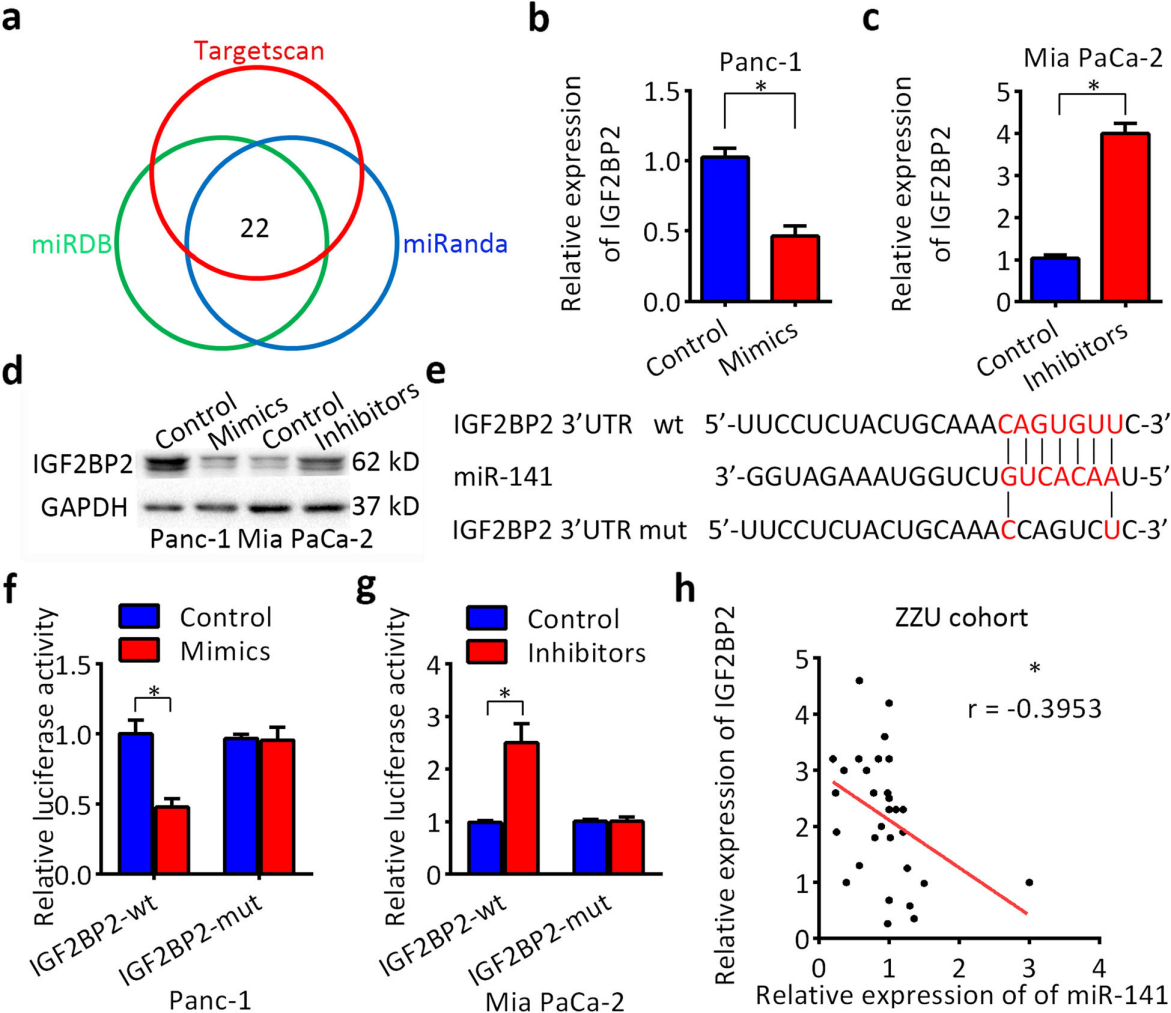
IGF2BP2 is a direct target of miR-141. **a** Intersections among three miRNA target prediction algorithms. **b** Schematic representation of IGF2BP2 3′-UTR indicating the predicted binding site of miR-141. **c-e** Relative expression of IGF2BP2 mRNA and protein after treatment with miR-141 mimics or inhibitors for 48 h. **f** and **g** Luciferase activity assays were performed to confirm the direct binding efficiency of miR-141 and its putative IGF2BP2 3′-UTR target. **h** Negative correlation between the expression levels of miR-141 and IGF2BP2 in PDAC tissues via Pearson’s correlation analysis. GAPDH served as a loading control. Data are presented as the mean ± SD of at least three independent measurements. *P < 0.05, **P < 0.01, and ***P < 0.001. *P* < 0.05 was considered statistically significant

microRNAs have been found to regulate gene expression posttranscriptionally, which prompted us to investigate whether the dysregulation of microRNAs contributes to IGF2BP2 upregulation. TargetScan, miRANDA and miRDB prediction algorithms were employed to identify potential microRNAs targeting IGF2BP2 (Fig. 4a). Among the 22 candidate miRNAs identified, miR-141 was frequently downregulated in 30 paired pancreatic cancer tissues (Additional file 10: Figure S6), so we decided to focus on the possibility that loss of miR-141 expression might promote the upregulation of IGF2BP2. Mimics or inhibitors of miR-141 were transfected into Panc-1 and Mia PaCa-2 cells, respectively. As shown in Fig. 4b-d, miR-141 dramatically inhibited the expression of IGF2BP2 at both the mRNA and protein levels. To investigate whether IGF2BP2 is a direct target of miR-141, a luciferase experiment was conducted. The sequences of the IGF2BP2 3′-UTR (wild-type or mutated) were cloned into a dual-luciferase reporter (Fig. 4e). Luciferase activity was significantly decreased by miR-141 mimics compared with that of the controls (Fig. 4f). In contrast, the luciferase activity was increased by miR-141 inhibitors (Fig. 4g). However, there were no effect on luciferase activity after transfection miR-141 mimics or inhibitors in the mutated IGF2BP2 3′-UTR group. Moreover, IGF2BP2 expression was inversely correlated with miR-141 expression in 30 pancreatic cancer tissues as detected by RT-qPCR (Fig. 4h). These results indicate that IGF2BP2 is regulated by miR-141 in PDAC, which is consistent with our hypothesis.

### IGF2BP2 activates the PI3K-Akt signaling pathway and promotes pancreatic cancer growth in vitro

**Fig. 5:**
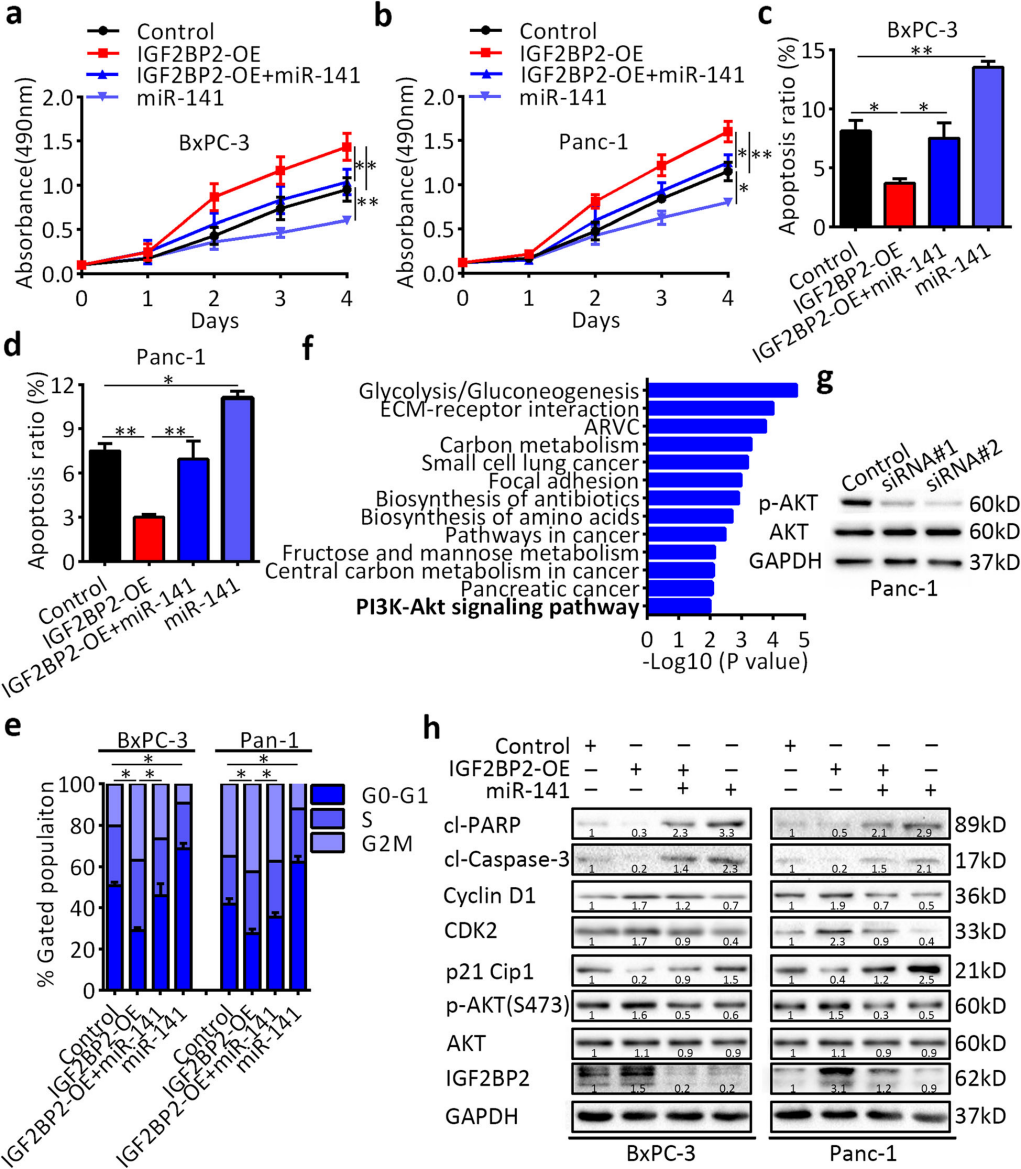
IGF2BP2 is a functional target of miR-141 in the miR-141-mediated suppression of pancreatic cancer cell proliferation. **a** and **b** The effect miR-141 transfection after IGF2BP2 overexpression on pancreatic cancer cell proliferation as determined by CCK8 assays. **c** and **d** Apoptosis analysis of IGF2BP2-overexpressing cells after miR-141 transfection by flow cytometry. **e** Cell cycle analysis of IGF2BP2-overexpressing cells after miR-141 transfection by flow cytometry. **f** Pathways associated with IGF2BP2 expression by KEGG enrichment analysis. **g** Expression of p-AKT by western blot analysis. **h** Expression of related regulators by western blot analysis. GAPDH served as a loading control. Data are presented as the mean ± SD of at least three independent measurements. *P < 0.05, **P < 0.01, and ***P < 0.001. *P* < 0.05 was considered statistically significant

To elucidate whether the miR-141 mediates the oncogenic activity of IGF2BP2, we first overexpressed IGF2BP2 in Panc-1 and BxPC-3 cells and then transfected them with miR-141 mimics. The expression of IGF2BP2 was verified by RT-qPCR analysis (Additional file 11: Figure S7). We found that overexpression of IGF2BP2 enhanced the proliferation ability of both cell lines, which was counteracted after miR-141 re-expression (Fig. 5a,b). Consistently, re-expression of miR-141 partly induced apoptosis (Fig. 5c,d), partially restoring the proportion of cells in the G0/G1 phase (Fig. 5e). It has been reported that IGF2BP2 promotes gene expression posttranscriptionally. Therefore, we analyzed the TCGA dataset and selected 207 genes that were positively correlated with IGF2BP2 expression (Poisson’s coefficient > 0.4). KEGG enrichment analysis showed that most of the genes were enriched in the PI3K-Akt pathway (Fig. 5f). Western blot analysis showed that the phosphorylated AKT(S473) level decreased after knockdown of IGF2BP2 in Panc-1 and BxPC-3 cells (Fig. 5g and Additional file 12: Figure S8). Furthermore, IGF2BP2 overexpression increased p-AKT expression in both Panc-1 and BxPC-3 cells (Fig. 5h). After miR-141 transfection, the levels of phosphorylated AKT(S473), CDK2, cyclin D1, cleaved caspase-3 and cleaved PARP were decreased, but p21 was increased. Western blot analysis further verified the previous findings. Taken together, these results indicate that the promotion of cell proliferation ability by IGF2BP2 is reversed to certain extents by the re-expression of miR-141, which is consistent with our hypothesis that IGF2BP2-mediated PI3K-Akt activation promotes pancreatic cancer growth.

### IGF2BP2 activates the PI3K-Akt signaling pathway and promotes pancreatic cancer growth in vivo

**Fig. 6:**
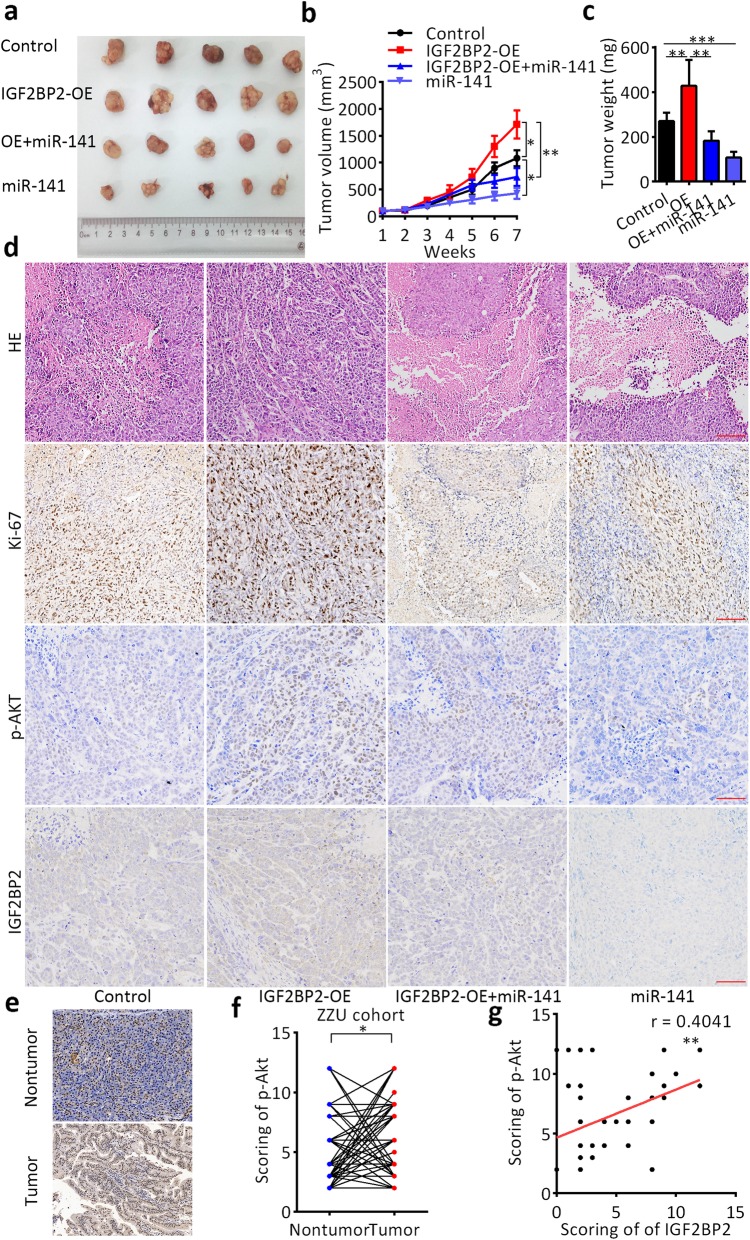
Promotion of pancreatic cancer growth by IGF2BP2 via PI3K-Akt signaling pathway activation in vivo. **a** Representative images of the subcutaneous xenografts of different treatment groups. **b** Tumor volume curves of different treatment groups. **c** Tumor weight of different treatment groups. **d** IHC staining of xenografts of different treatment groups. Scale bar, 50 μm (red line). **e** Representative images of phosphorylated AKT(S473) staining in human pancreatic cancer tissues and matched nontumor tissues. Scale bar, 50 μm (red line). **f** Relative expression score of phosphorylated AKT(S473) in pancreatic cancer by IHC. **g** Positive correlation between the expression level of IGF2BP2 and level of phosphorylated AKT(S473) by IHC in human pancreatic cancer tissues. *P < 0.05, **P < 0.01, and ***P < 0.001. *P* < 0.05 was considered statistically significant

To determine whether IGF2BP2 promotes pancreatic cancer cell growth via the PI3K-Akt pathway in vivo as observed in vitro, we established a subcutaneous xenograft model with different treatments (Fig. 6a). As shown in Fig. 6b and c, cells overexpressing IGF2BP2 formed larger tumors than control cells formed. However, miR-141 re-expression partially blocked the tumor-promoting effect of IGF2BP2. IHC staining of the tissues resected from the xenograft tumors revealed that positive staining of the proliferation marker Ki-67 and phosphorylated AKT(S473) staining were increased in the IGF2BP2 overexpression group, but decreased in the miR-141 re-expression groups (Fig. 6d). In addition, p-Akt(S473) staining was also performed on 60 pairs of pancreatic cancer tissues and nontumor tissues. Quantitative analysis indicated that p-Akt(S473) staining was increased in human PDAC tissues compared with nontumor tissues (Fig. 6e,f; *n* = 60). In addition, the p-Akt(S473) level was also positively correlated with the IGF2BP2 expression level (Fig. 6g; *n* = 60). We also stained the cell cycle and apoptosis markers CDK2, cyclin D1, p21, and cleaved PARP, the trend changes of which were similar to those observed in vitro by western blot (Additional file 13: Figure S9). These results suggest that the PI3K-Akt signaling pathway is essential for the oncogenic role of IGF2BP2 in pancreatic cancer both in vitro and in vivo.

### Downregulation of miR-141 correlates with a poor survival of pancreatic cancer patients

**Fig. 7:**
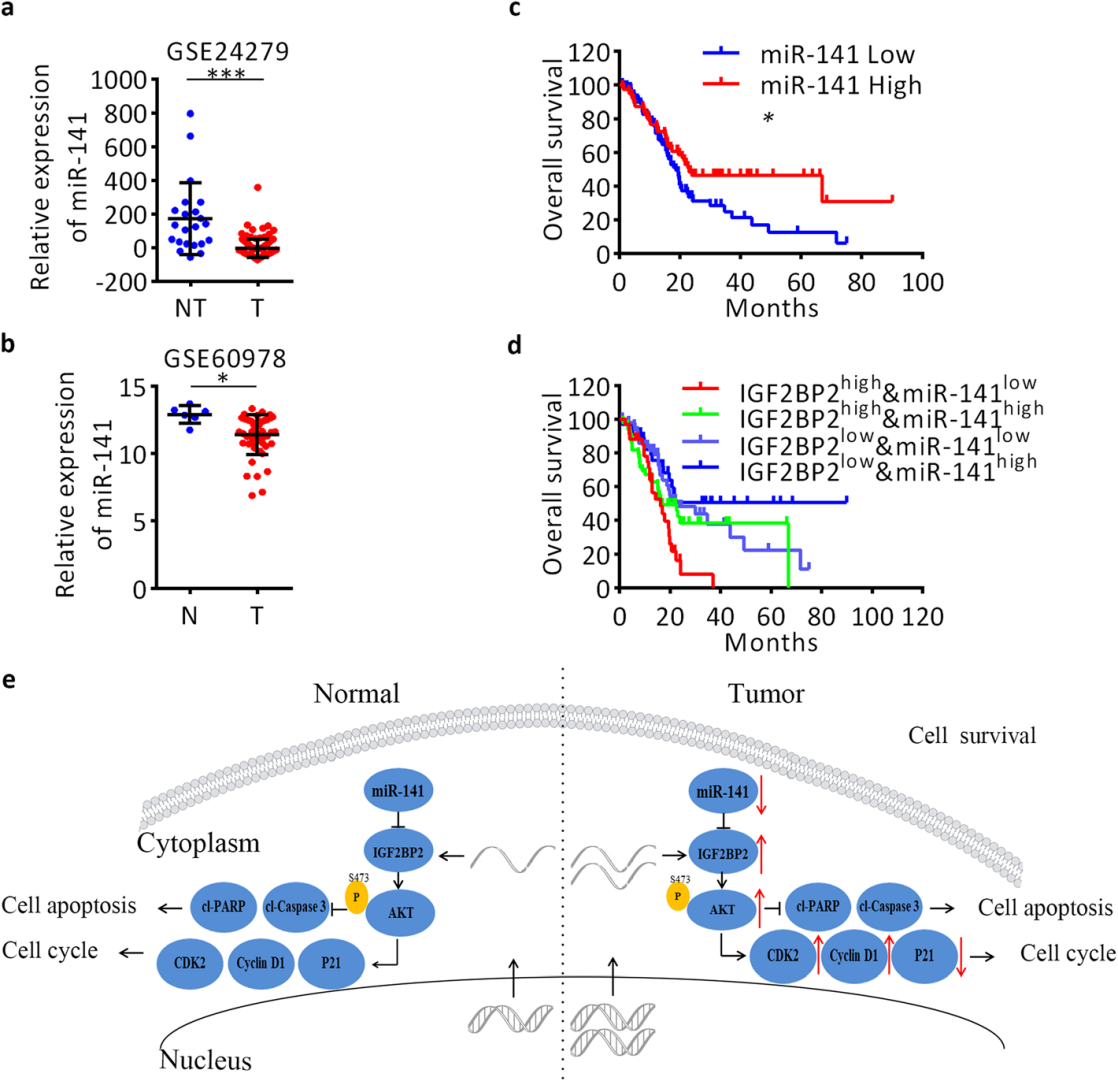
Low expression of miR-141 correlates with worse clinical outcome in pancreatic cancer patients. **a** and **b** Expression of miR-141 in pancreatic cancer based on GEO datasets. **c** Survival analysis of miR-141 based on the TCGA dataset. **d** Survival curves of IGF2BP2^high^&miR-141^low^, IGF2BP2^high^&miR-141^high^, IGF2BP2^low^&miR-141^low^ and IGF2BP2^low^& miR-141^high^ groups. The median survival times for each group were 16.36 months, 16.79 months, 22.83 months and undefined, respectively. **e** Illustration of overexpression of IGF2BP2 by multiple mechanisms activates PI3K-Akt signaling in pancreatic cancer cells. U6 served as an internal reference. Data are presented as the mean ± SD of at least three independent measurements. *P < 0.05, **P < 0.01, and ***P < 0.001. *P* < 0.05 was considered statistically significant

Based on the GEO datasets, we found that miR-141 expression is significantly downregulated in pancreatic cancer tissues compared to normal controls (Fig. 7a, b), which is Consistent with the RT-qPCR detection in 30 paired pancreatic cancer tissues. To determine the clinical significance of miR-141 in pancreatic cancer, we mined the TCGA dataset. Kaplan–Meier survival analysis of the TCGA dataset showed that patients with low expression levels of miR-141 displayed shorter overall survival than patients with high miR-141 expression (median: 18.66 months vs. 23.06 months, respectively; log-rank test, *P* < 0.05; Fig. 7c). To determine whether miR-141 is an independent predictive factor of survival, a Cox multivariate regression analysis was performed. The results showed that miR-141 maintained independence when predicting the OS of patients with pancreatic cancer (HR = 0.368, 95% CI =0.255–0.785, *P* < 0.001; Additional file 4: Table S4). Furthermore, based on the expression levels of both miR-141 and IGF2BP2, we classified 171 patients in the TCGA dataset into four groups. As shown in Fig. 7d, patients with IGF2BP2^high^&miR-141^low^ levels displayed significantly poorer survival than patients with IGF2BP2^low^&miR-141^high^ levels (median: 16.36 months vs. undefined, respectively; log-rank test, *P* < 0.01). Taken together, these results suggest that miR-141 may represent a novel biomarker for pancreatic cancer and that a combination of IGF2BP2 and miR-141 can improve the prediction of outcomes in patients with pancreatic cancer.

## Discussion

Previous studies have demonstrated that IGF2BP2 participates in the initiation and progression of numerous cancers. However, the biological functions and molecular mechanisms of IGF2BP2 in pancreatic cancer remain elusive. In this study, we found that IGF2BP2 was overexpressed in pancreatic cancer and predicted poor clinical outcomes, and IGF2BP2 promoted oncogenesis by activating the PI3K-Akt signaling pathway. In addition, the present study identified that genomic alterations and loss of miR-141 partly contribute to IGF2BP2 overexpression and facilitate the oncogenic effect of IGF2BP2 in pancreatic cancer.

A previous study revealed that IGF2BP2 was upregulated in ovarian serous carcinoma effusions and associated with poor survival [19]. Based on the TCGA dataset, we found that IGF2BP2 was effective in stratifying pancreatic cancer patients into high and low-risk of death groups. ROC curve analysis revealed that AUC values performed well in predicting the disease course in patients with pancreatic cancer. IGF2BP2 might be helpful for identifying patients who need aggressive and individualized therapies to improve their clinical outcomes.

To further study the mechanism of IGF2BP2 upregulation in pancreatic cancer, we first investigated the genomic amplification variation and gene expression level of IGF2BP2 the TCGA PADC dataset. However, high IGF2BP2 mRNA upregulation rate with a relative low genomic copy number gain rate suggested that other regulation mechanisms might play important roles in IGF2BP2 overexpression in pancreatic cancer. Therefore, we focused on the posttranscriptional regulation mechanisms, such as dysregulation of miRNAs. After bioinformatic analysis and experimental validation, miR-141 was confirmed to directly regulate IGF2BP2, and its expression showed a negative correlation with IGF2BP2 expression in primary samples. Rescue experiments showed that IGF2BP2 is a functional target of miR-141 in pancreatic cancer because miR-141 re-expression partially reversed the tumor-promotive effect of IGF2BP2. Studies have also reported that downregulation of miR-141 inhibited the development and progression of colorectal cancer and gastric adenocarcinoma [20, 21]. It has been reported that miR-141 is downregulated in a genetically engineered KrasG12D; Pdx1-Cre mouse (KC) model during tumor progression [22]. Hypermethylation of the miR-141 promoter regions induces miR-200c downregulation [23], indicating that epigenetic alterations might induce the loss of miR-141. Therefore, altered methylation of miR-141 promoter regions decreases miR-141 expression levels and enhances IGF2BP2 expression and enhances tumor progression of pancreatic cancer. Accordingly, our results showed that pancreatic cancer patients with IGF2BP2^high^&miR-141^low^ had the worst survival outcome, while patients with IGF2BP2^low^&miR-141^high^ showed longest survival time.

A recent study on pancreatic cancer also demonstrated that upregulation of IGF2BP2 predicted a poor prognosis and was associated with metastasis [24]. However, the function of IGF2BP2 on proliferation of pancreatic cancer cells was analyzed in our research. The flow cytometry assays further indicated that IGF2BP2 inhibited cell apoptosis and induced cell cycle progression in pancreatic cancer. Additionally, the GSEA analysis demonstrated that IGF2BP2 promotes cell cycle progression, which further demonstrates the pro-proliferative characteristics of IGF2BP2. IGF2BP2 mediates stabilization of HMGA1 mRNA and production of IGF2 which synergistically drive cancer progression [12]. IGF2BP2 activates the small Rho-GTPase RAC1 and induces ROS production which activates NADPH oxidase in human HCC [25]. However, we found that overexpression of IGF2BP2 induced Ser473 phosphorylation of Akt which suggests that IGF2BP2 promotes pancreatic cancer cell survival via activation of the Akt signaling pathway (Fig. 7e).

## Conclusions

In summary, IGF2BP2 is frequently upregulated in pancreatic cancer and the expression of IGF2BP2 is regulated by genomic alterations and the silencing of miR-141. IGF2BP2 promotes pancreatic cancer growth by activating the PI3K-Akt signaling pathway. Our data suggest that IGF2BP2 may serve as a new therapeutic target for patients with pancreatic cancer.

## Supplementary information

**Additional file 1: Table S1.** Primers and sequences used in this research (5′-3′).

**Additional file 2: Table S2.** Multivariable Cox regression analysis of OS in pancreatic cancer patients in the ZZU cohort.

**Additional file 3: Table S3.** Multivariable Cox regression analysis of OS in pancreatic cancer patients in the TCGA dataset.

**Additional file 4: Table S4.** Multivariable Cox regression analysis of OS in pancreatic cancer patients in the TCGA dataset.

**Additional file 5: Figure S1.** Expression of IGF2BP2 in TCGA pancreatic cancer tissues (*n* = 171).

**Additional file 6: Figure S2.** Expression of IGF2BP2 in TCGA pancreatic cancer tissues and normal tissues.

**Additional file 7: Figure S3.** Receiver operating characteristic analysis of the sensitivity and specificity of the overall survival prediction by the the expression of IGF2BP2.

**Additional file 8: Figure S4.** Relative expression of IGF2BP2 in pancreatic cancer cells after transfection.

**Additional file 9: Figure S5.** Flow cytometry assay of apoptosis of BxPC-3 and Panc-1 cells after transfection.

**Additional file 10: Figure S6.** Expression of miR-141 in PDAC tissues and adjacent noncancerous tissues by miRNA RT-qPCR.

**Additional file 11: Figure S7.** Relative expression of IGF2BP2 in pancreatic cancer cells after transfection.

**Additional file 12: Figure S8.** Western blot analysis of the phosphorylated AKT(S473) levels after knockdown of IGF2BP2 in BxPC-3 cells.

**Additional file 13: Figure S9.** IHC staining of xenografts of different treatment groups.

## Data Availability

All data in our study are available upon request.

## Abbreviations

CCK-8: Cell counting kit 8
CI: Confidence interval
CNAs: Copy number alterations
CNV: Coby numer variation
DMEM: Dulbecco’s Modified Eagle’s Medium
FBS: Fetal bovine serum
GEO: Gene Expression Omnibus
HR: Hazard ratio
IGF2BP2: IGF2 mRNA-binding protein 2
IHC: Immunohistochemistry
OS: Overall survival
PBS: Phosphate buffered solution
PDAC: Pancreatic ductal carcinoma
PVDF: Polyvinyli denedifluoride
ROC: Receiver operating characteristic
RPMI-1640: Roswell Park Memorial Institute 1640
RT-qPCR: Real-time quantitative polymerase chain reaction
SD: Standard deviations
SDS-PAGE: Sodium dodecyl sulfate-poly acrylamide gel electrophoresis
TCGA: The Cancer Genome Atlas
