# Supplementary material for: Up-regulation of IGF2BP2 by multiple mechanisms in pancreatic cancer promotes cancer proliferation by activating the PI3K/Akt signaling pathway
Source: J Exp Clin Cancer Res. 2019 Dec 18;38:497. doi: 10.1186/s13046-019-1470-y (PMC6921559; doi:10.1186/s13046-019-1470-y)
Supplement: Supplementary file 1 — Additional file 1: Table S1. Primers and sequences used in this research (5′-3′). [file 13046_2019_1470_MOESM1_ESM.docx]

**Table S1.** Primers and sequences used in this research (5’-3’).

| GAPDH forward | CTCACCGGATGCACCAATGTT |
| --- | --- |
| GAPDH reverse | CGCGTTGCTCACAATGTTCAT |
| IGF2BP2 forward | AGTGGAATTGCATGGGAAAATCA |
| IGF2BP2 reverse | CAACGGCGGTTTCTGTGTC |
| hsa-miR-141 forward | TCTCGCCTTCTTTCCCATCTTT |
| hsa-miR-141 reverse | CCGCTAACACTGTCTGGTAAAG |
| U6 forward | CTCGCTTCGGCAGCACA |
| U6 reverse | AACGCTTCACGAATTTGCGT |
| IGF2BP2 siRNA#1 | AGTGAAGCTGGAAGCGCATAT |
| IGF2BP2 siRNA#2 | TTCCCGCATCATCACTCTTAT |
