# Supplementary material for: Up-regulation of IGF2BP2 by multiple mechanisms in pancreatic cancer promotes cancer proliferation by activating the PI3K/Akt signaling pathway
Source: J Exp Clin Cancer Res. 2019 Dec 18;38:497. doi: 10.1186/s13046-019-1470-y (PMC6921559; doi:10.1186/s13046-019-1470-y)
Supplement: Supplementary file 2 — Additional file 2: Table S2. Multivariable Cox regression analysis of OS in pancreatic cancer patients in the ZZU cohort. [file 13046_2019_1470_MOESM2_ESM.docx]

**Table S2.** Multivariable Cox regression analysis of OS in pancreatic cancer patients in the ZZU cohort.

| Characteristic | | Multivariable analysis | | | |
| --- | --- | --- | --- | --- | --- |
|  |  | | HR | 95% CI | P-value |
| Sex | Female/Male | | 1.270 | 0.669-2.410 | 0.465 |
| Age, years | ≥65 /<65 | | 1.436 | 0.739-2.792 | 0.286 |
| Tumor stage | III.IV/I.II | | 0.931 | 0.115-7.502 | 0.946 |
| Tumor grade | III.IV/I.II | | 1.587 | 0.797-3.159 | 0.189 |
| TNM stage | III.IV/I.II | | 1.454 | 0.502-4.209 | 0.490 |
| Size | ≥2.5cm/<5cm | | 0.984 | 0.422-2.294 | 0.970 |
| IGF2BP2 | High/Low | | 2.395 | 1.655-4.134 | <0.05 |
