# Supplementary material for: Up-regulation of IGF2BP2 by multiple mechanisms in pancreatic cancer promotes cancer proliferation by activating the PI3K/Akt signaling pathway
Source: J Exp Clin Cancer Res. 2019 Dec 18;38:497. doi: 10.1186/s13046-019-1470-y (PMC6921559; doi:10.1186/s13046-019-1470-y)
Supplement: Supplementary file 4 — Additional file 4: Table S4. Multivariable Cox regression analysis of OS in pancreatic cancer patients in the TCGA dataset. [file 13046_2019_1470_MOESM4_ESM.docx]

**Table S4.** Multivariable Cox regression analysis of OS in pancreatic cancer patients in the TCGA dataset.

| Characteristic | | Multivariable analysis | | | |
| --- | --- | --- | --- | --- | --- |
|  |  | | HR | 95% CI of HR | P-value |
| Sex | Female/Male | | 1.310 | 0.706-2.63 | 0.389 |
| Age, years | ≥65 /<65 | | 1.526 | 0.812-2.869 | 0.186 |
| TNM | III.IV/I.II | | 0.954 | 0.325-3.502 | 0.946 |
| Tumor grade | III.IV/I.II | | 1.587 | 0.797-3.159 | 0.189 |
| Size, cm | ≥2.5/<2.5 | | 1.654 | 0.896-3.105 | <0.01 |
| miR-141 | High/Low | | 0.368 | 0.255-0.785 | <0.001 |
