# Supplementary figures and images for: Up-regulation of IGF2BP2 by multiple mechanisms in pancreatic cancer promotes cancer proliferation by activating the PI3K/Akt signaling pathway
Source: J Exp Clin Cancer Res. 2019 Dec 18;38:497. doi: 10.1186/s13046-019-1470-y (PMC6921559; doi:10.1186/s13046-019-1470-y)

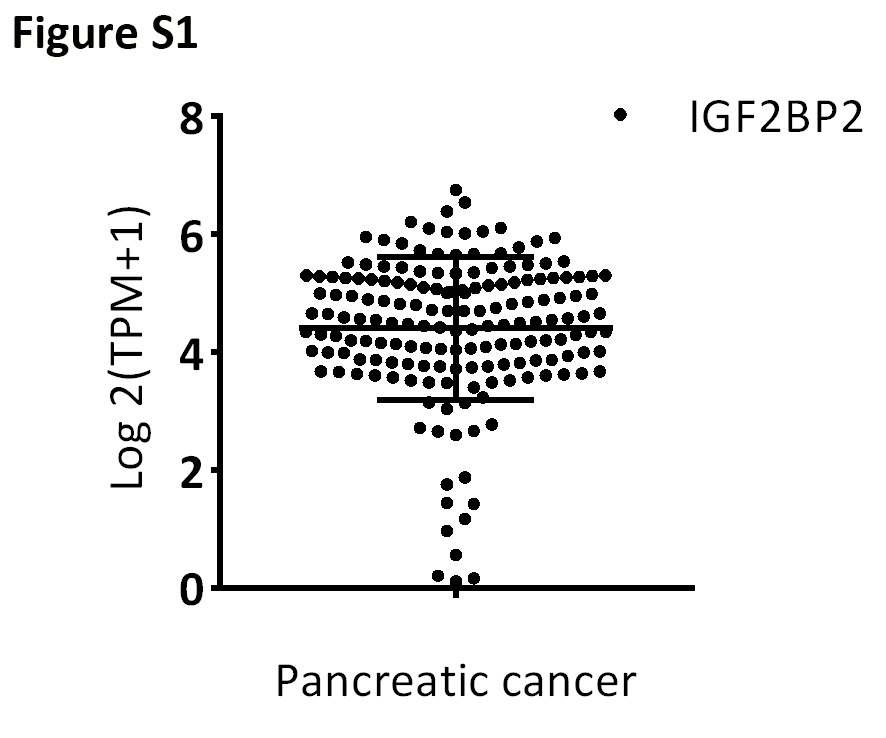

Supplement: Supplementary file 5 — Additional file 5: Figure S1. Expression of IGF2BP2 in TCGA pancreatic cancer tissues (n = 171). [file 13046_2019_1470_MOESM5_ESM.tif]

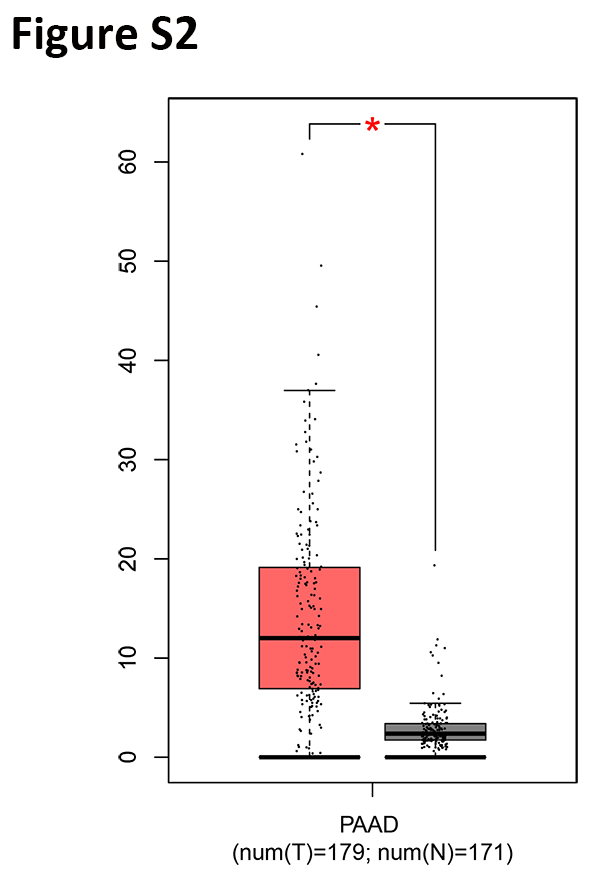

Supplement: Supplementary file 6 — Additional file 6: Figure S2. Expression of IGF2BP2 in TCGA pancreatic cancer tissues and normal tissues. [file 13046_2019_1470_MOESM6_ESM.tif]

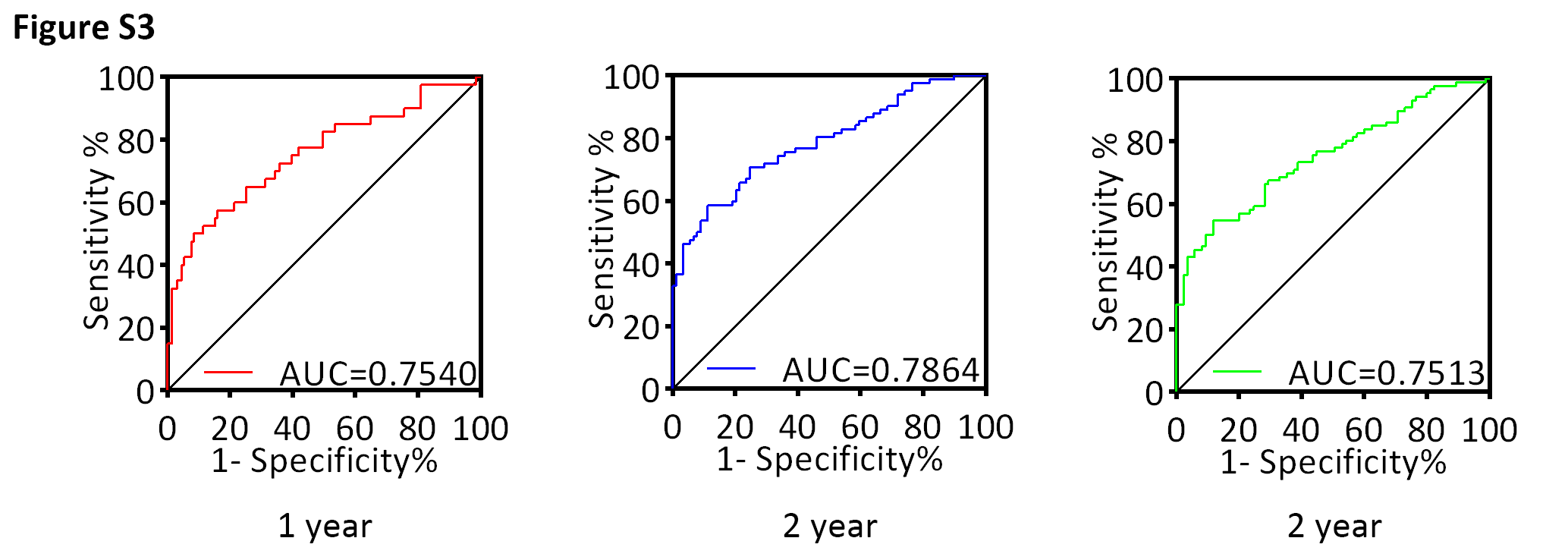

Supplement: Supplementary file 7 — Additional file 7: Figure S3. Receiver operating characteristic analysis of the sensitivity and specificity of the overall survival prediction by the the expression of IGF2BP2. [file 13046_2019_1470_MOESM7_ESM.tif]

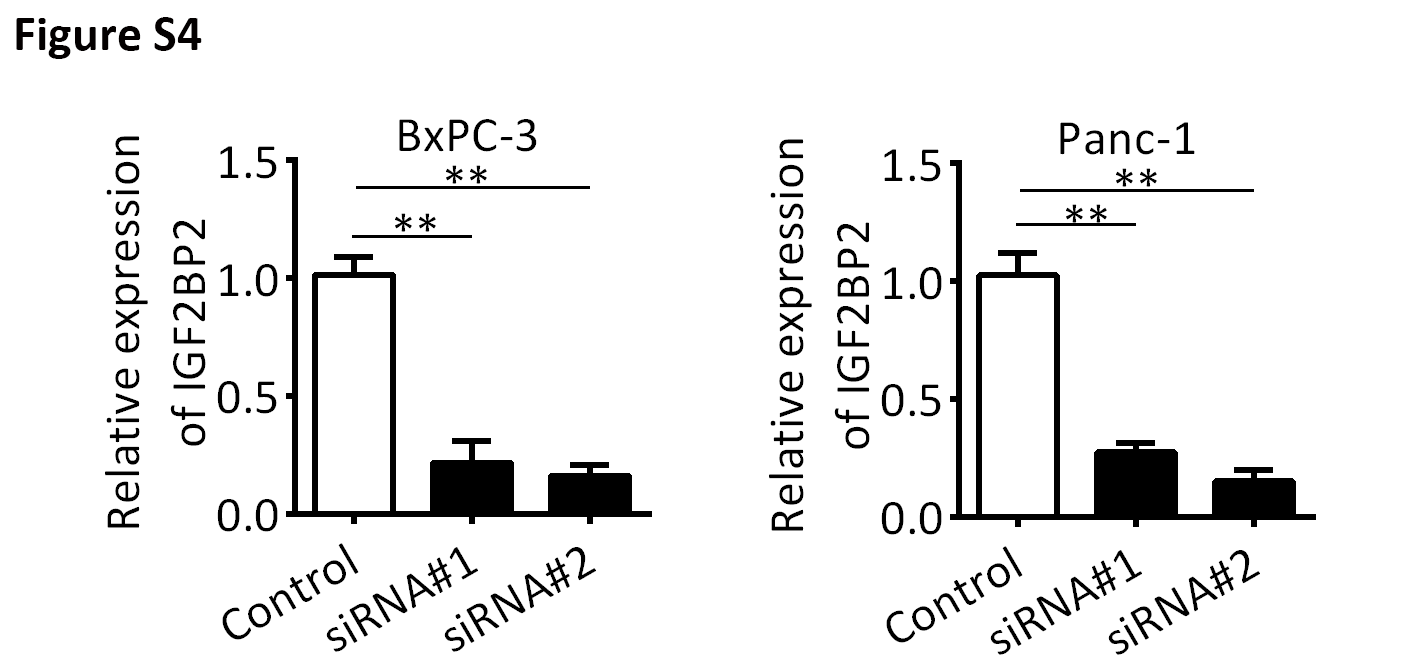

Supplement: Supplementary file 8 — Additional file 8: Figure S4. Relative expression of IGF2BP2 in pancreatic cancer cells after transfection. [file 13046_2019_1470_MOESM8_ESM.tif]

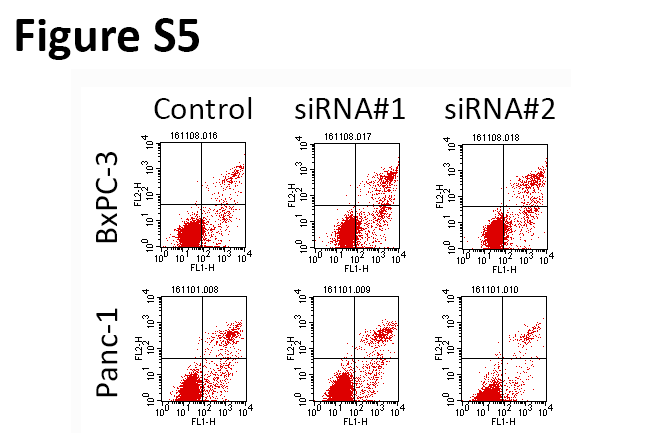

Supplement: Supplementary file 9 — Additional file 9: Figure S5. Flow cytometry assay of apoptosis of BxPC-3 and Panc-1 cells after transfection. [file 13046_2019_1470_MOESM9_ESM.tif]

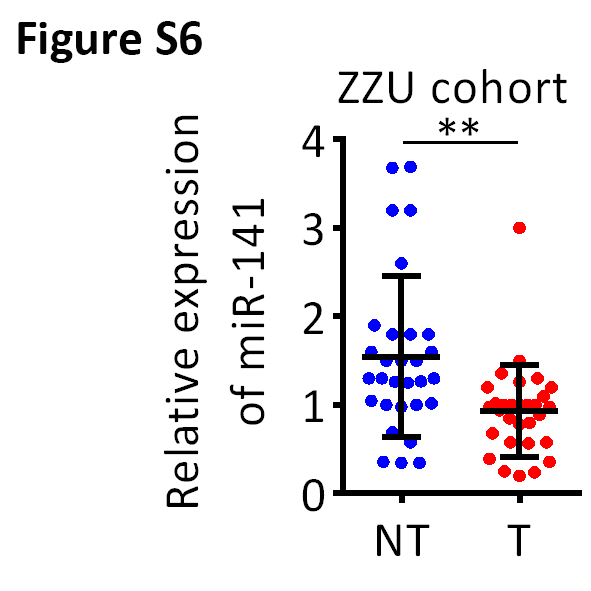

Supplement: Supplementary file 10 — Additional file 10: Figure S6. Expression of miR-141 in PDAC tissues and adjacent noncancerous tissues by miRNA RT-qPCR. [file 13046_2019_1470_MOESM10_ESM.tif]

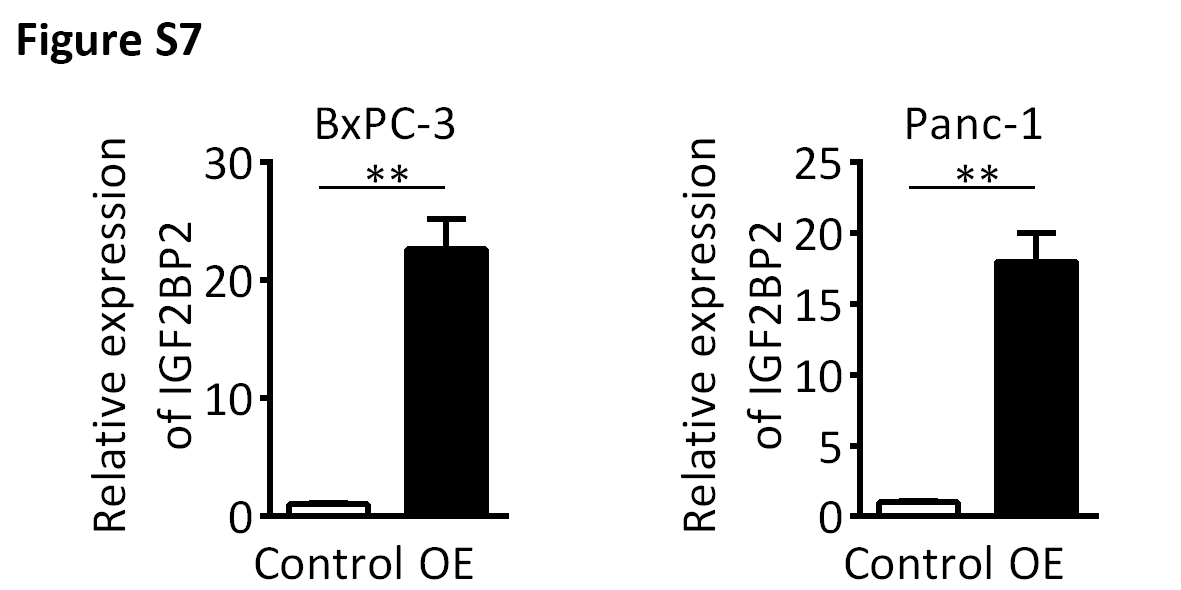

Supplement: Supplementary file 11 — Additional file 11: Figure S7. Relative expression of IGF2BP2 in pancreatic cancer cells after transfection. [file 13046_2019_1470_MOESM11_ESM.tif]

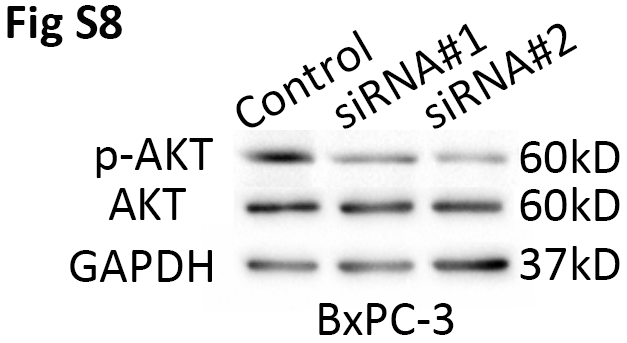

Supplement: Supplementary file 12 — Additional file 12: Figure S8. Western blot analysis of the phosphorylated AKT(S473) levels after knockdown of IGF2BP2 in BxPC-3 cells. [file 13046_2019_1470_MOESM12_ESM.tif]

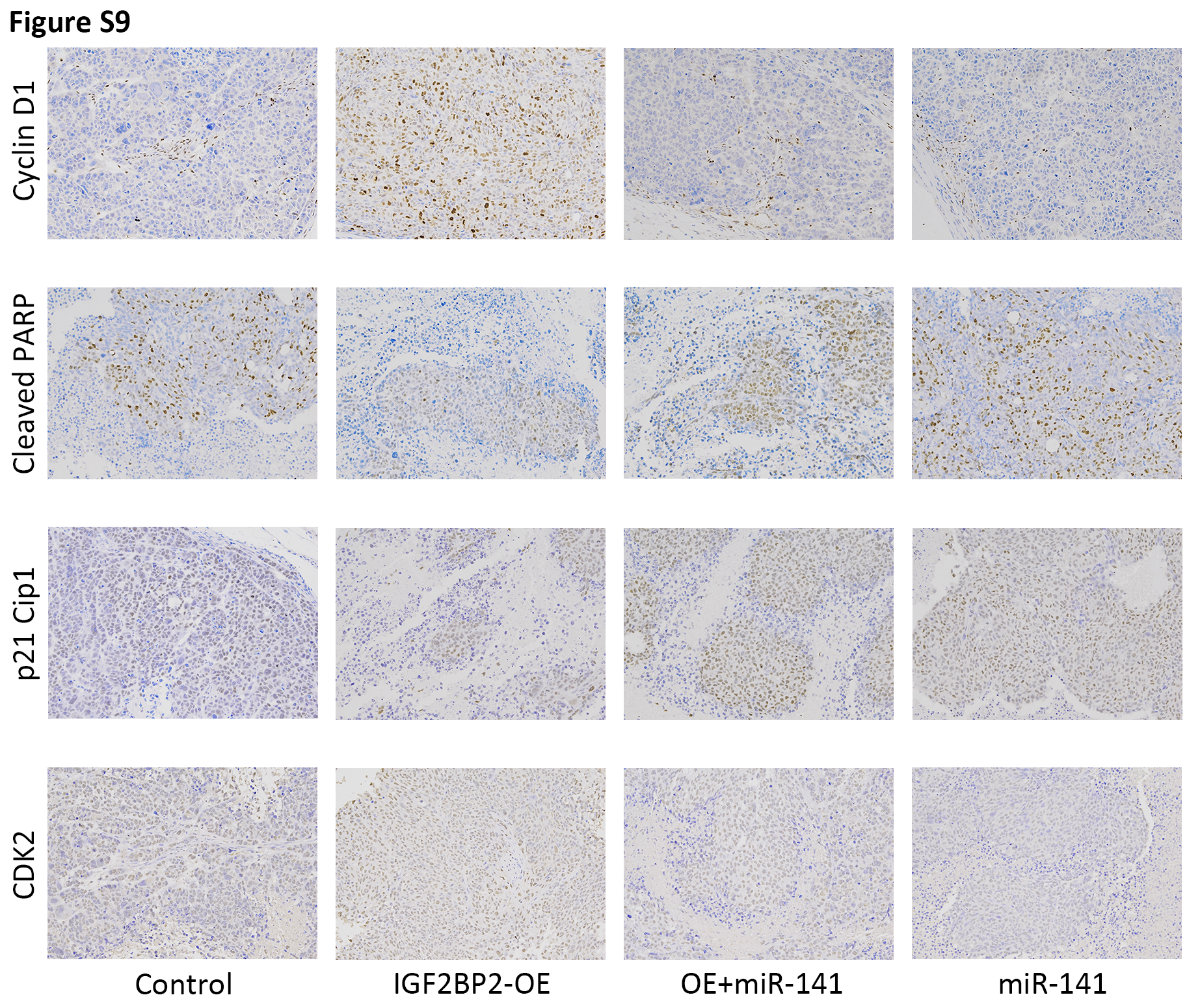

Supplement: Supplementary file 13 — Additional file 13: Figure S9. IHC staining of xenografts of different treatment groups. [file 13046_2019_1470_MOESM13_ESM.tif]
